# Serological Assessment of *Ascaris suum* Exposure in Greek Pig Farms and Associated Risk Factors Including *Lawsonia intracellularis*

**DOI:** 10.3390/pathogens11090959

**Published:** 2022-08-23

**Authors:** Panagiotis Tassis, Isaia Symeonidou, Athanasios I. Gelasakis, Michalis Kargaridis, George Aretis, Konstantinos V. Arsenopoulos, Eleni Tzika, Elias Papadopoulos

**Affiliations:** 1 Farm Animals Clinic, School of Veterinary Medicine, Faculty of Health Sciences, Aristotle University of Thessaloniki, 54627 Thessaloniki, Greece; 2 Laboratory of Parasitology and Parasitic Diseases, School of Veterinary Medicine, Faculty of Health Sciences, Aristotle University of Thessaloniki, 54124 Thessaloniki, Greece; 3 Laboratory of Anatomy and Physiology of Farm Animals, Department of Animal Science, School of Animal Biosciences, Agricultural University of Athens, 11855 Athens, Greece; 4 Gerolymatos International SA, 13 Asklipiou St., Kryoneri, 14568 Athens, Greece; 5 Boehringer Ingelheim Hellas, Leof. Andrea Siggrou 340, Kallithea, 17673 Athens, Greece

**Keywords:** *Ascaris suum*, *Lawsonia intracellularis*, serology, risk factors, Greece, swine farms

## Abstract

The effects of nematodes and bacteria on intestinal health are of primary importance in modern swine production. The aim of the present study was to assess the seropositivity status of *Ascaris suum* infection in fatteners in intensive swine farms in Greece and address possible risk factors, including *Lawsonia intracellularis* as a predisposing factor to swine ascariosis. In total, 360 blood serum samples from pigs in the late fattening period, from 24 Greek swine farrow-to-finish farms (15 samples/farm) were collected and tested with Svanovir^®^ *A. suum* antibody ELISA and Ileitis antibody ELISA. The results demonstrated 34.4% seropositive samples for *A. suum* and 42.2% for *L. intracellularis.* The analysis of predisposing risk factors suggested that the frequency of application of anthelminthic treatment to sows more than two times per year was significantly associated with the lower likelihood of *A. suum* infection, whereas a greater likelihood of *A. suum* infection was observed in pigs with concurrent *L. intracellularis* exposure. The results highlight the importance of proper anthelminthic metaphylaxis of the breeding stock, as well as the likely outcome of concurrent exposure to two intestinal pathogens in pigs, implying a possible association between intestinal nematodes and bacteria in swine.

## 1. Introduction

Modern feeding strategies and pig management tools require proper intestinal health, maturation and adaptation to feeding alterations through the production life of swine. Intestinal homeostasis plays a critical role in the health and productivity of swine. Pathogens affecting the intestinal balance provoke typical enteric diseases and clinical symptoms (e.g., diarrhea, reduced growth, etc.) as well as nutrient imbalance, thus compromising health and reducing the profitability of swine farms globally [1,2,3].

Pigs may harbor quite a few intestinal parasites, nematodes and protozoa. One of the most common gastrointestinal nematodes with a global distribution is *Ascaris suum* [4]. This parasitic roundworm occurs in all kinds of pig production systems and may affect all age groups [4,5]. Although the course of porcine ascariosis is usually subclinical, clinical infections may occur in growing pigs [6]. Furthermore, the hepato-tracheal migration of *A. suum* larvae results in injury and subsequent inflammation (white or milk spots) leading to the induction of respiratory distress characterized by frequent dry coughing or even severe dyspnea in some animals [5]. In addition, parasitized pigs become more susceptible to infections and diseases, which sabotage their health and welfare [7]. Decreased nutritional conversion and average daily body mass gain are hallmarks of *A. suum* infection [5,8] and a linear correlation between worm burdens and these two parameters has been established [9]. Additionally, the presence of white spots in the liver of pigs leads to the condemnation of affected organs in abattoirs, thus reducing the carcass value [10]. All of the above, taken together, document that porcine ascariosis can result in major economic losses [11,12], whereas *A. suum* may also have public health implications [4,13].

Given the above, the knowledge of the prevalence and intensity of *A. suum* infection is crucial for evaluating the success of the implemented control measures, for tailoring management practices such as housing and applied antiparasitic treatments and for determining the economic impact of the infection. In modern conventional units, ascariosis is not commonly diagnosed. The diagnosis of *A. suum* infection is challenging due to its subclinical nature in combination with the lack of sensitive diagnostic tools [14]. Post-mortem assessments of infected organs and fecal egg counts have certain limitations; thus, the actual infection rate and effects of *A. suum* on the pigs’ health and productivity are often underestimated. Towards this end, serology can provide more accurate information both on the infection pressure to which animals are exposed and on its impact on farm sustainability [14,15]. 

In addition, the recognition of specific risk factors is essential for prevention and for improving targeted control schemes [16]. Age [17] as well as outdoor rearing under organic farming conditions [18] have been previously assessed as potential risk factors for *A. suum* infection. It has also been proposed that concurrent intestinal pathogens such as the obligate intracellular bacterium *Lawsonia intracellularis* might have an additional negative influence on pig performance [5]. *Lawsonia intracellularis* has been proven to affect intestinal function in swine, inducing porcine proliferative enteropathy (PPE) [19]. Three clinically different forms of PPE are more commonly observed: acute (young adults 4–12 months of age); chronic (weaned pigs 6–20 weeks of age); and subclinical [20]. 

To date, in Greece, there has only been one recent multicenter study on the occurrence of intestinal parasites in intensively reared pigs that recorded a mean prevalence of 3.7% for *A. suum* by coprological examination [17]. However, data regarding the seropositivity of *A. suum* infection that imply a challenge with this parasite are lacking.

The objectives of this cross-sectional study were (i) to assess the seropositivity status of *A. suum* infection in fatteners in intensive swine farms in Greece, that has been formed under the commonly applied antiparasitic schemes in Greek intensive farms in the last few decades, and (ii) to assess the possible risk factors, including for the first time the potential implication of *L. intracellularis*, which could predispose to porcine ascariosis in intensive units.

## 2. Results

### 2.1. Seropositivity of Ascaris suum in the Studied Population

A cut-off optical density ratio (ODr) value of 0.50 was used for the presentation of results, according to Vlaminck et al. [21]. In total, 124 out of 360 samples (34.4%) were positive. The majority of farms (18/24 farms) had at least one positive sample, whereas the greatest incidence level was observed in one farm with all samples testing positive. The mean ODr values of the positive samples in total were 0.74 (± 0.35 standard deviation (SD)). Evaluation of the regional positivity pattern showed that 34% of the samples from the northern region were positive (mean ODr: 0.65 ± 0.15 SD); 28.9% were positive in the south (ODr: 0.69 ± 0.17); 40% were in the central part of the country (ODr: 0.80 ± 0.52); and 34.4% in the west (ODr: 0.81 ± 0.33). The absence of significant differences between the positive samples’ percentages and the mean ODr levels among regions was reported (*p* = 0.482). 

### 2.2. Seropositivity of Lawsonia Intracellularis in the Studied Population

In total, 152 out of 360 samples (42.2%) were positive with a mean percent inhibition (PI) value of 47.42 (±19.73 SD). In 3 out of the 24 farms, all samples were positive, whereas all samples were negative for one farm. The results showed that 31.1% in the north (PI: 51.50 ± 19.44), 53.5% in the south (PI: 53.62 ± 21.22), 25.6% in the central region (PI: 36.05 ± 17.47) and 58.9% of samples in the west (PI: 47.08 ± 16.64) were positive. Mean PI values of the central region showed a reduced proportion of positive cases with PI > 30% in comparison to all other regions (*p* = 0.001).

### 2.3. Effects of Risk Factors on Ascaris suum Seropositivity in Farms

The random intercept variance for the unconditional mean model was 1.87±0.754 (*p* ≤ 0.05, CI 95% 0.85 to 4.12), and the ICC was equal to 0.36 indicating that 36% of the likelihood of *A. suum* infection was being explained by between-farm differences, while the rest, 64%, was attributable to within-farm factors. The application of anthelmintic treatment in pigs was not significantly associated with the presence of *A. suum* infection on the studied farms. A lower likelihood of infection with *A. suum* tended to occur on farms practicing anthelmintic treatment in sows >2 times per year compared to the farms that treated the sows less frequently (decreased by ca. 3.8 times, *p* = 0.052, CI 95%, 0.99 to 14.92 times). On the other hand, a significantly higher likelihood of infection with *A. suum* tended to occur in pigs with *L. intracellularis* co-infection when compared to non-co-infected animals (increased by ca. 2.1 times, *p* = 0.040, CI 95%, 1.04 to 4.29 times). The effects of the studied predictors used in the multilevel binary regression model (PAT, ATF and LIC) are summarized below (Table 1).

## 3. Discussion

*Ascaris suum* is the most prevalent helminth in swine farms worldwide [11,22], though its prevalence differs depending on management factors and geographical areas [18,23]. Commonly, low prevalence and infection intensity are recorded in intensive swine production, while high levels of ascariosis are recorded in traditional and organic swine farms [23,24]. Moreover, management practices applied on individual farms determine the infection pattern among different age groups [17].

The estimation of the prevalence of *A. suum* infection is of major importance. The usefulness of serology has been evaluated and this method indeed provides a more precise estimation of the prevalence of ascariosis [12,15,21,25]. Vlaminck et al. [26] noted that the level of seropositivity for *A. suum* is linked to pig performance and can be used to reflect infection intensity. Based on our serology results, seroconversion due to *A. suum* infection in fatteners was 34.4%. A recent multicenter extensive study in Greece employing coprological methods recorded a prevalence of 0.9% for *A. suum* in fatteners in conventional intensive Greek pig farms [17]. This discrepancy in results is expected because in fecal tests, false-negative results are common. This has been attributed to the presence of immature or single-sex worms and the aggregation of adults [14] as well as to the fact that a large proportion of infected pigs do not excrete eggs in their feces despite positive serological response results [27]. An additional explanation is that serological results represent previous infection weeks prior to sampling, whilst fecal tests performed in our previous study display the level of *A. suum* occurrence in feces at the time of sampling. The recorded seroprevalence rate in the present study is somewhere between the respective values observed in outdoor Iberian fattening pigs (23.8–35%) [28] and in fatteners from northern Spain (48%) [15]. However, it is lower than the assessed mean seroprevalence of fattening pigs in Sichuan Province in China, which was a striking >60% [29].

Our results are the first to show the extent of *L. intracellularis* seroprevalence (42.2%) in Greek farms. It has been demonstrated that *L. intracellularis* infection can result in reduced growth upwards of 60% [30]. Furthermore, it has been recorded that increased PPE incidence can reduce farm profitability as evidenced in respective studies in German [31], Danish [32] and UK farms [33]. Acute cases are characterized by sudden death with anemia and hemorrhagic diarrhea, whereas pigs affected by the chronic form display mild to moderate loose to watery grey-green diarrhea, anorexia and reduced growth [20]. Since none of the farms included in our study had increased morbidity levels or mortality (data not presented) due to ileitis, it can be suggested that significant seroconversion on particular farms can be associated with the subacute form of the disease. According to previous studies, the detection of antibodies indicates an exposure to *L. intracellularis* in the past, but does not provide clear information about current infections in the animals [19]. Previous evidence on seroconversion in an experimental study was described within two weeks after exposure [34]. Antibodies against *L. intracellularis* may be present until slaughter age as also seen in our study [34,35]. A large-scale study in five European countries detected at least one seropositive sample against *L. intracellularis* in 91.7% of the sampled herds (60 herds) [19]. Quite similarly in our study, 95.83% of the tested farms had at least one case of seroconversion due to *L. intracellularis*. These findings confirm the wide distribution of the pathogen in Greek farms, in accordance with other European countries.

Significant findings of the present study have been highlighted by the binary regression model results regarding the risk factors of *A. suum* seropositivity. In total, in-feed anthelminthic substances for the metaphylaxis of internal parasitism in fatteners were used in 10 out of 24 study farms. Six out of ten farms were administering ivermectin and four farms fenbendazole at the age of approximately 10 weeks. Surprisingly, the application of anthelmintic treatment in pigs was not significantly associated with *A. suum* infection in the studied farms. The absence of a significant seroconversion discrepancy among farms administering anthelminthic substances and those who do not, may rely on multiple factors such as the chronic or subacute infection status of the farms. Furthermore, the proper treatment of sows could result in the disruption of the *A. suum* life cycle and reduction in in-farm transmission dynamics (e.g., reduced eggs shedding in farrowing facilities), thus minimizing the risk of infection to fatteners. Since metaphylactic anthelminthic treatments for sows were applied in the aforementioned 10 farms, such evidence indicates that the prophylaxis of the fatteners against nematodes through the proper anthelminthic metaphylaxis of sows is a possible scenario.

The beneficial effect of applying anthelminthic treatments more than two times per year to the breeding stock of farms has been confirmed for ascariosis in the present risk factor analysis. In particular, infections were almost 4 times less likely to occur when sows were treated regularly at less than 6-month intervals. Likewise, a nearly 5 times lower probability of another helminth infection caused by *Trichuris* *suis* (*T. suis*) has been previously suggested when anthelmintic drugs were administered regularly at less than 4-month intervals in sows [17]. These results indicate that such administration schemes for sows fed under field conditions should be practiced. In humans, the recommended frequency of treatment for high-risk communities (more than 50% prevalence of ascariosis) is twice per year and for low-risk communities (infection prevalence between 20% and 50%) it is once per year [36]. Both in humans and in pigs, in cases of a high prevalence of infection, repeated anthelmintic treatment is highly recommended.

Our results suggested an approximate two-fold greater likelihood of *A. suum* infection when *L. intracellularis* is present in the herd. Similarly, a study demonstrated a significant correlation between the number of pigs that were positive for *L. intracellularis* by PCR and the number of *T. suis* eggs found in grower and finisher feces, as well as the number of *A. suum* eggs found in finisher feces under commercial conditions. The association at the herd level between infection with *L. intracellularis* and the presence of *T. suis* was robust since whipworm-positive herds were 17.43 times more likely to also be positive for *L. intracellularis* [37]. A synergistic effect between *T. suis* and *L. intracellularis* has been previously described regarding necrotizing proliferative colitis and the reduction in performance in growing pigs [38]. This effect has been attributed to the interplay via mutual immunosuppression between intestinal nematodes and enteric bacteria [37]. Nevertheless, the evidence of the increased impact on diarrhea and performance due to concurrent *A. suum* and *L. intracellularis* infection, as suggested by Boes et al. [5], in combination with our findings highlighting the increased likelihood of *A. suum* incidence when *L. intracellularis* is present, suggest that awareness should be increased when interpreting diarrhea cases at the fattening stage, especially in farms where anthelminthic treatments are not applied after weaning. In the same context, in foals it has been proposed that intestinal parasitism could make them more susceptible to *L. intracellularis* infection [39]. Additionally, it has been established that *L. intracellularis* can affect the immune response by reducing numbers of B and T cell populations in heavily infected pigs, enable persistent infection of crypt epithelial cells and alter the composition of gut microbiota (GM). The above may increase the probability of co-infections with other bacteria, such as the case of increased colonization and shedding of *Salmonella enterica* [20,40,41,42].

Furthermore, enhanced pathogenicity or susceptibility to viral or bacterial infections, associated with the immune-modulatory and migratory capacity of *A. suum* has been reported and this should not be ignored [11]. This indirect consequence on the pigs’ health is the outcome of the modulation of the immunity of the host by the nematode with the induction of a strong polarized T-helper (Th)-2-type immune response, which suppresses the necessary Th1-driven immunity for combating bacterial and viral infections [43]. Indeed, it has been evidenced that the presence of *A. suum* enhances the effects of infections with *Escherichia coli* and *Pasteurella multocida* [44,45]. The damage caused in the lungs can pave the way for opportunistic bacterial and viral infections [45]. Moreover, *A. suum*-mediated changes in GM composition may result in impaired nutrient metabolism and immunity to concurrent infections with other pathogens. Subsequently, a study under field conditions has shown that when piglets are infected with the nematodes *A. suum* and *T. suis,* they excrete more *Campylobacter* spp. [46]. Another experiment investigating infection reported that infected pigs have a thorough change in the diversity of the colon microbiome including an increase in *Prevotella* and a reduction in *Ruminococcus* populations along with the suppression of their metabolic pathways concerning amino acid and carbohydrate acid metabolism [47]. The aforementioned research confirms that *A. suum* infection may challenge the composition and resiliency of the GM not only to its predilection site of infection (the small intestine), but also in other distal organs, i.e., the colon and caecum. Furthermore, a possible interference with vaccination has been proposed for this nematode although the exact immunosuppressive mechanisms for this interplay are not fully clarified [48,49]. It has been demonstrated that concurrent *A. suum* infections can impair the effect of vaccination against *Mycoplasma hyopneumoniae* in pigs [48]. Combining the above-mentioned facts with results of the present study, the need for further investigation of similar co-occurrence patterns and interactions of *A. suum* with other enteric pathogens at the intestinal immune response level is emphasized.

## 4. Materials and Methods

### 4.1. Sampling Process

To assess *A. suum* and *L. intracellularis* exposure of Greek pig farms, 24 farrow-to-finish farms were tested and 15 blood samples were collected from pigs in the late fattening period (>18 weeks of age), either at the fattening unit or at the slaughterhouse, from each farm. The total number of sows under production in the selected farms exceeded 20% of the total sow population reared in intensive farrow-to-finish farms in Greece.

The farms were geographically located in four different areas of Greece, namely the northern (Macedonia region), central (Thessaly region), southern (Evia, Viotia and Peloponnese region) and western (Epirus and Aitoloakarnania region) regions. Six farms per region were included in the study. Detailed information was recorded from the sampled farms to investigate the risk factors involved. The following data were recorded: the implementation of anthelminthic treatment in fatteners and sows (type, dosage level, timing, substance, frequency of administration, age of fatteners), the antibody levels against *L. intracellularis* and the implementation of treatment for *L. intracellularis* (type, dosage level, timing, substance). 

Among them, the application of anthelmintic treatment in pigs (PAT), the frequency of anthelmintic treatment in sows (ATF) and the co-infection with *L. intracellularis* (LIC) were retained as the most appropriate ones to be assessed as risk factors in the studied farms considering the reliability of the collected information and the variation between the farms. 

### 4.2. Serological Methods

Initially, samples were collected with a vacutainer system (18G 1¼ inch needle) from the jugular vein in BD vacutainer tubes (10 mL). Samples were thereafter centrifuged (3000 rpm × 10 min) and serum was collected and stored (−18 °C) until analysis. 

Samples were tested with the use of Svanovir^®^ *A. suum* antibody ELISA and Svanovir^®^ *L. intracellularis*/Ileitis-antibody ELISA (blocking ELISA based on whole cell *L. intracellularis* antigen), as per the manufacturer’s instructions (Boehringer Ingelheim Svanova), for the assessment of antibodies against *A. suum* and *L. intracellularis*, respectively (analytical steps and controls presented by Vlaminck et al. [21] and Jacobson et al. [50], respectively). The Svanovir^®^ *A. suum*-Ab assay is an indirect ELISA based on the hemoglobin (AsHb) of this roundworm, thus enabling the detection of antibodies to both larval and adult stages. Reactivity to the AsHb antigen is shown in ODr (ODr sample = (ODsample–ODnegative control)/(ODpositive control–ODnegative control) [21]. The diagnostic specificity and sensitivity of Svanovir^®^ *A. suum* antibody ELISA is 100% for experimentally infected animals and under field conditions it remains high [21]. Likewise, for the Ileitis antibody ELISA, specificity is 93% and sensitivity is 72% (at cut-off PI value of 35) [50]. All serological tests were performed in Bioscreen laboratory (Hannover, Germany). 

According to manufacturer’s instructions, *A. suum* ELISA ODr values < 0.4 showed absence or a low-level exposure to *A. suum*, values between 0.4 and 0.6 suggested possible exposure but limited impact of the parasite, whilst values exceeding 0.6 demonstrated significant exposure to *A. suum*. Regarding *L. intracellularis*, ELISA results were considered to be negative when the inhibition levels were <20%, ambiguous at levels ranging from 20 to 30% and positive at levels exceeding 30%.

### 4.3. Data Handling—Statistical Analyses

A multilevel (two-level) binary logistic regression model was used. Pigs (level 1) were nested within the farms (level 2) to test the possible relationship between the PAT, ATF and LIC and the likelihood that a pig is infected with *A. suum*, after adjusting for the random effect of the farm. 

Initially, an unconditional mean model was used to estimate the intraclass correlation coefficient (ICC) and quantify the proportion of the between-farm variation in the total variation, as below [51]

$$\mathrm{ICC}=\frac{var\left(u0j\right)}{var\left(u0j\right)+\left(\pi^{2}/3\right)}$$

where ICC is the intraclass correlation coefficient, *var(u*0*j)* is the random intercept variance and *π*^2^/3 (≈3.29) is the standard logistic distribution (assumed level-1 variance component).

Afterwards, a full model containing the main effects of the level-1 predictors and both level-1 and level-2 residuals was developed as described below

Yij = B0 + (B1 + u1j) × PATij + (B2 + u2j) × ATFij + (B3 + u3j) × LICij + uj

where Yij is the odds of a pig i within the farm j being infected with *A. suum*; B0 is the fixed intercept; B1 to B3 are the fixed coefficients of a) the application of the anthelmintic treatment of pigs (PAT) (2 levels: the application of anthelmintic treatment and no application of anthelmintic treatment), the anthelmintic treatment frequency in the sows (ATF) (2 levels: ≤2 times per year and >2 times per year) and *L. intracellularis* co-infection (LIC) (3 levels: definite co-infection, ambiguous co-infection and no co-infection); u1j to u3j are the residual terms associated with the predictors used in the model, namely, PAT, ATF and LIC (u1j to u3j); and uj is the random residual (associated with the farm). 

## 5. Conclusions

Serology is a promising tool for diagnosing ascariοsis at the fattening stage. To the best of our knowledge, this is the first time the seroprevalence of *A. suum* and *L. intracellularis* of pigs reared in farrow-to-finish conventional intensive Greek farms has been assessed. The results of *A. suum* serological screening highlight that this infection in commercial pig farms in Greece is largely underestimated, thus encouraging future large-scale studies to clarify the actual extent of *A. suum* exposure and infection and to therefore contribute to implementing effective control schemes for porcine ascariosis. Moreover, the present study underlines the importance of conducting anthelminthic treatments more than two times per year in sows and indicates the increased probability of *A. suum* infection under the effect of concurrent *L. intracellularis* presence on fatteners. Coinfection dynamics and the effects between *A. suum* and bacteria that could impair the intestinal function and performance of pigs should be further investigated. 

## Figures and Tables

**Table 1:** Coefficients ± standard errors, p-values and odds ratios (with 95% confidence intervals) of the studied predictors (PAT, ATF and LIC) forced into the multilevel binary regression model to assess their association with the likelihood of *Ascaris suum* infection.

	B	S.E.	P	Odds Ratio	95% C.I. for EXP(B)	
					Lower	Upper
PAT	−0.33	0.651	0.612	0.72	0.20	2.58
No PAT	Ref.					
ATF ≤ 2 times per year	1.35	0.69	0.052	3.84	0.99	14.92
ATF > 2 times per year	Ref.					
Definite LIC	0.75	0.362	0.040	2.11	1.04	4.29
Ambiguous LIC	0.15	0.449	0.748	1.16	0.48	2.80
No LIC	Ref.					

B: coefficients, S.E.: standard errors, C.I.: confidence interval, PAT: application of anthelmintic treatment in pigs, ATF: frequency of anthelmintic treatment in sows, LIC: *Lawsonia intracellularis* co-infection.

## Data Availability

The data presented in this study are available on request from the corresponding author.

## Abbreviations

PPE: porcine proliferative enteropathy; PAT: application of anthelmintic treatment in pigs; ATF: anthelmintic treatment frequency in sows; LIC: *Lawsonia intracellularis* co-infection; ODr: optical density ratio; PI: percent inhibition; GM: gut microbiota.

## References

[1] Everaert N., Van Cruchten S., Weström B., Bailey M., Van Ginneken C., Thymann T., Pieper R. (2017). A review on early gut maturation and colonization in pigs, including biological and dietary factors affecting gut homeostasis. Anim. Feed Sci. Technol..

[2] Thomson J.R., Friendship R.M., Zimmerman J., Karriker L.A., Ramirez A., Schwartz K.J., Stevenson G.W., Zhang J. (2019). Digestive system. Diseases of Swine.

[3] Modina S.C., Aidos L., Rossi R., Pocar P., Corino C., Di Giancamillo A. (2021). Stages of Gut Development as a Useful Tool to Prevent Gut Alterations in Piglets. Animals.

[4] Roepstorff A., Mejer H., Nejsum P., Thamsborg S.M. (2011). Helminth parasites in pigs: New challenges in pig production and current research highlights. Vet. Parasitol..

[5] Boes J., Kanora A., Havn K.T., Christiansen S., Vestergaard-Nielsen K., Jacobs J., Alban L. (2010). Effect of *Ascaris suum* infection on performance of fattening pigs. Vet. Parasitol..

[6] Joachim A., Dülmer N., Daugschies A., Roepstorff A. (2001). Occurrence of helminths in pig fattening units with different management systems in Northern Germany. Vet. Parasitol..

[7] Brewer M.T., Greve J.H., Zimmerman J.J., Karriker L.A., Ramirez A., Schwartz K.J., Stevenson G.W., Zhang J. (2019). Internal Parasites. Diseases of Swine.

[8] Lassen B., Oliviero C., Orro T., Jukola E., Laurila T., Haimi-Hakala M., Heinonen M. (2017). Effect of fenbendazole in water on pigs infected with *Ascaris suum* in finishing pigs under field conditions. Vet. Parasitol..

[9] Hale O.M., Stewart T.B., Marti O.G. (1985). Influence of an experimental infection of *Ascaris suum* on performance of pigs. J. Anim. Sci..

[10] Pedersen S., Saeed I., Michaelsen K.F., Friis H., Murrell K.D. (2002). Impact of protein energy malnutrition on *Trichuris suis* infection in pigs concomitantly infected with *Ascaris suum*. Parasitology.

[11] Thamsborg S.M., Nejsum P., Mejer H., Holland C. (2013). Impact of *Ascaris suum* in livestock. Ascaris: The Neglected Parasite.

[12] Vandekerckhove E. (2018). The use of serology in the control of *Ascaris suum* infections in pigs. Ph.D. Thesis.

[13] Nejsum P., Betson M., Bendall R.P., Thamsborg S.M., Stothard J.R. (2012). Assessing the zoonotic potential of *Ascaris suum* and *Trichuris suis*: Looking to the future from an analysis of the past. J. Helminthol..

[14] Vlaminck J., Levecke B., Vercruysse J., Geldhof P. (2014). Advances in the diagnosis of *Ascaris suum* infections in pigs and their possible applications in humans. Parasitology.

[15] Martínez-Pérez J.M., Vandekerckhove E., Vlaminck J., Geldhof P., Martínez-Valladares M. (2017). Serological detection of *Ascaris suum* at fattening pig farms is linked with performance and management indices. Vet. Parasitol..

[16] Fontanarrosa M.F., Vezzani D., Basabe J., Eiras D.F. (2006). An epidemiological study of gastrointestinal parasites of dogs from Southern Greater Buenos Aires (Argentina): Age, gender, breed, mixed infections, and seasonal and spatial patterns. Vet. Parasitol..

[17] Symeonidou I., Tassis P., Gelasakis A.Ι., Tzika E.D., Papadopoulos E. (2020). Prevalence and risk factors of intestinal parasite infections in Greek swine farrow-to-finish farms. Pathogens.

[18] Kochanowski M., Karamon J., Dąbrowska J., Dors A., Czyżewska-Dors E., Cencek T. (2017). Occurrence of intestinal parasites in pigs in Poland—The influence of factors related to the production system. J. Vet. Res..

[19] Arnold M., Crienen A., Swam H., von Berg S., Jolie R., Nathues H. (2019). Prevalence of *Lawsonia intracellularis* in pig herds in different European countries. Porc. Health Manag..

[20] Vannucci F.A., Gebhart C.J., McOrist S., Zimmerman J., Karriker L.A., Ramirez A., Schwartz K.J., Stevenson G.W., Zhang J. (2019). Proliferative Enteropathy. Diseases of Swine.

[21] Vlaminck J., Nejsum P., Vangroenweghe F., Thamsborg S.M., Vercruysse J., Geldhof P. (2012). Evaluation of a serodiagnostic test using *Ascaris suum* haemoglobin for the detection of roundworm infections in pig populations. Vet. Parasitol..

[22] Dold C., Holland C.V. (2011). Ascaris and ascariasis. Microbes Infect..

[23] Katakam K.K., Thamsborg S.M., Dalsgaard A., Kyvsgaard N.C., Mejer H. (2016). Environmental contamination and transmission of *Ascaris suum* in Danish organic pig farms. Parasit. Vectors.

[24] Raue K., Heuer L., Böhm C., Wolken S., Epe C., Strube C. (2017). 10-year parasitological examination results (2003 to 2012) of faecal samples from horses, ruminants, pigs, dogs, cats, rabbits and hedgehogs. Parasitol. Res..

[25] Joachim A., Winkler C., Ruczizka U., Ladinig A., Koch M., Tichy A., Schwarz L. (2021). Comparison of different detection methods for *Ascaris suum* infection on Austrian swine farms. Porc. Health Manag..

[26] Vlaminck J., Düsseldorf S., Heres L., Geldhof P. (2015). Serological examination of fattening pigs reveals associations between *Ascaris suum*, lung pathogens and technical performance parameters. Vet. Parasitol..

[27] Roepstorff A. (1998). Natural *Ascaris suum* infections in swine diagnosed by coprological and serological (ELISA) methods. Parasitol. Res..

[28] Sánchez-Murillo J.M. (2003). Epidemiología de la ascariosis porcina en Extremadura. Ph.D. Thesis.

[29] Zheng Y., Xie Y., Geldhof P., Vlaminck J., Ma G., Gasser R.B., Wang T. (2020). High anti-*Ascaris* seroprevalence in fattening pigs in Sichuan, China, calls for improved management strategies. Parasit. Vectors..

[30] Helm E.T., Burrough E.R., Leite F.L., Gabler N.K. (2021). *Lawsonia intracellularis* infected enterocytes lack sucrase-isomaltase which contributes to reduced pig digestive capacity. Vet. Res..

[31] Schoelen A. (2007). Untersuchungen zu den ökonomischen Auswirkungen der Porzinen Proliferativen Enteropathie in der Schweinemast. Ph.D. Thesis.

[32] Jensen H.M. (2006). Health Management with Reduced Antibiotic Use—Experiences of a Danish Pig Vet. Anim. Biotechnol..

[33] McOrist S., Smith S.H., Green L.E. (1997). Estimate of direct financial losses due to porcine proliferative enteropathy. Vet Rec..

[34] Guedes R.M.C., Gebhart C.J. (2003). Onset and duration of fecal shedding, cell-mediated and humoral immune responses in pigs after challenge with a pathogenic isolate or attenuated vaccine strain of *Lawsonia intracellularis*. Vet. Microbiol..

[35] Stege H., Jensen T.K., Møller K., Vestergaard K., Baekbo P., Jorsal S.E. (2004). Infection dynamics of *Lawsonia intracellularis* in pig herds. Vet. Microbiol..

[36] World Health Organization (2011). Helminth Control in School-Age Children: A Guide for Managers of Control Programmes.

[37] Pearce G.P. (1999). Interactions between dietary fibre, endo-parasites and *Lawsonia intracellularis* bacteria in grower–finisher pigs. Vet. Parasitol..

[38] Mansfield L.S., Urban J.F. (1996). The pathogenesis of necrotic proliferative colitis in swine is linked to whipworm induced suppression of mucosal immunity to resident bacteria. Vet. Immunol. Immunopathol..

[39] Bohlin A.M., Olsen S.N., Laursen H.S., Öhman A., van Galen G. (2019). *Lawsonia intracellularis* associated equine proliferative enteropathy in Danish weanling foals. Acta Vet. Scand..

[40] Macintyre N., Smith D.G.E., Shaw D.J., Thomson J.R., Rhind S.M. (2003). Immunopathogenesis of experimentally induced proliferative enteropathy in pigs. Vet. Pathol..

[41] Beloeil P.A., Fravalo P., Fablet C., Jolly J.P., Eveno E., Hascoet Y., Chauvin C., Salvat G., Madec F. (2004). Risk factors for *Salmonella enterica subsp. enterica* shedding by market-age pigs in French farrow-to-finish herds. Prev. Vet. Med..

[42] Leite F.L.L., Singer R.S., Ward T., Gebhart C.J., Isaacson R.E. (2018). Vaccination against *Lawsonia intracellularis* decreases shedding of *Salmonella enterica* serovar *Typhimurium* in co-infected pigs and alters the gut microbiome. Sci. Rep..

[43] Zakeri A., Hansen E.P., Andersen S.D., Williams A.R., Nejsum P. (2018). Immunomodulation by helminths: Intracellular pathways and extracellular vesicles. Front. Immunol..

[44] Adedeji S.O., Ogunba E.O., Dipeolu O.O. (1989). Synergistic effect of migrating *Ascaris suum* larvae and *Escherichia coli* in piglets. J. Helminthol..

[45] Tjørnehøj K., Eriksen L., Aalbaek B., Nansen P. (1992). Interaction between *Ascaris suum* and *Pasteurella multocida* in the lungs of mice. Parasitol. Res..

[46] Jensen A.N., Mejer H., Mølbak L., Langkjær M., Jensen T.K., Angen Ø., Martinussen T., Klitgaard K., Baggesen D.L., Thamsborg S.M. (2011). The effect of a diet with fructan-rich chicory roots on intestinal helminths and microbiota with special focus on Bifidobacteria and *Campylobacter* in piglets around weaning. Animal.

[47] Wang Y., Liu F., Urban J.F., Paerewijck O., Geldhof P., Li R.W. (2019). *Ascaris suum* infection was associated with a worm-independent reduction in microbial diversity and altered metabolic potential in the porcine gut microbiome. Int. J. Parasitol..

[48] Steenhard N.R., Jungersen G., Kokotovic B., Beshah E., Dawson H.D., Urban J.F., Roepstorff A., Thamsborg S.M. (2009). Ascaris suum infection negatively affects the response to a *Mycoplasma hyopneumoniae* vaccination and subsequent challenge infection in pigs. Vaccine.

[49] Williams A.R., Myhill L.J., Stolzenbach S., Nejsum P., Mejer H., Nielsen D.S., Thamsborg S.M. (2021). Emerging interactions between diet, gastrointestinal helminth infection, and the gut microbiota in livestock. BMC Vet. Res..

[50] Jacobson M., Wallgren P., Nordengrahn A., Merza M., Emanuelson U. (2011). Evaluation of a blocking ELISA for the detection of antibodies against *Lawsonia intracellularis* in pig sera. Acta Vet. Scand..

[51] Sommet N., Morselli D. (2017). Keep calm and learn multilevel logistic modeling: A simplified three-step procedure using stata, R, Mplus and SPSS. Int. Rev. Soc. Psychol..

